# Measuring discrimination- and reversal learning in mouse models within 4 days and without prior food deprivation

**DOI:** 10.1101/lm.042085.116

**Published:** 2016-11

**Authors:** Esther Remmelink, August B. Smit, Matthijs Verhage, Maarten Loos

**Affiliations:** 1 Sylics (Synaptologics B.V.), 1008 BA Amsterdam, The Netherlands; 2 Department of Molecular and Cellular Neurobiology, Center for Neurogenomics and Cognitive Research, Neuroscience Campus Amsterdam, VU University, 1081 HV Amsterdam, The Netherlands; 3 Department of Functional Genomics, Center for Neurogenomics and Cognitive Research, Neuroscience Campus Amsterdam, VU University, 1081 HV Amsterdam, The Netherlands

## Abstract

Many neurological and psychiatric disorders are characterized by deficits in cognitive flexibility. Modeling cognitive flexibility in mice enables the investigation of mechanisms underlying these deficits. The majority of currently available behavioral tests targeting this cognitive domain are reversal learning tasks that require scheduled food restriction, extended training periods and labor-intensive, and stress-inducing animal handling. Here, we describe a novel 4-day (4-d) continuously running task measuring discrimination- and reversal learning in an automated home cage (CognitionWall DL/RL task) that largely eliminates these limitations. In this task, mice can earn unlimited number of food rewards by passing through the correct hole of the three-holed CognitionWall. To assess the validity and sensitivity of this novel task, the performance of C57BL/6J mice, amyloid precursor protein/presenilin1 transgenic (APP/PS1) mice, α-calmodulin kinase-II (αCaMKII) T305D knock-in mice, and mice with an orbitofrontal cortex lesion were examined. We found that C57BL/6J mice reach stable performance levels within the 4 d of the task, while experiencing only slight reductions in weight and no major effects on circadian rhythm. The task detected learning deficits in APP/PS1 transgenic and αCaMKII T305D mutant mice. Additionally, we established that the orbitofrontal cortex underlies reversal learning performance in our task. Because of its short duration and the absence of food deprivation and concurrent weight loss, this novel automated home-cage task substantially improves comprehensive preclinical assessment of cognitive functions in mouse models of psychiatric and neurological disorders and also enables analysis during specific developmental stages.

In many brain disorders, including schizophrenia (Murray et al. 2008; Leeson et al. 2009) and autism (D'Cruz et al. 2013), the balance between automatic and goal-directed responding is shifted toward less flexible behavioral patterns. Tasks frequently applied to measure behavioral- or cognitive flexibility in humans have the same underlying principle: to respond adequately and flexibly to an unexpected change in a learned stimulus–reward association to maximize reward.

Many rodent analogs of human tasks have been developed (Brigman et al. 2010), where the most used paradigms involve operant chambers in which mice have to learn to poke (Laughlin et al. 2011), press (Ortega et al. 2013), or touch (Brigman et al. 2005) to obtain a food or liquid reward. During initial discrimination learning (DL), when mice learn to discriminate between unrewarded and rewarded response options, valuable information is gathered on associative learning abilities. Once mice have reached stable performance levels during DL, the reward associations are reversed during a reversal learning (RL) phase, or changed toward a different stimulus dimension during a shift phase, to measure the ability of mice to flexibly adjust responses accordingly.

These operant procedures require daily periods of total food deprivation until the next feeding moment to maintain body weight at 90%–80% of original, prior to and during the entire training period of the task, in order to keep mice motivated to work for rewards. Although mild food restriction and/or intermittent feeding may have benefits to rodent physiology, morbidity, and lifespan (Martin et al. 2010), food deprivation is generally considered a stressor (Heiderstadt et al. 2000; Guarnieri et al. 2012) that should ideally be minimized. Moreover, food deprivation may affect behavioral responses differentially in different mouse strains and models (Cabib et al. 2000; Conrad 2010; Papaleo et al. 2012), potentially impacting on results obtained in mice. In addition, operant protocols for reversal learning can take up to 40 d for mice to reach the performance criteria (Mar et al. 2013), partly because they rely on the less developed mouse visual system. Ideally, these lengthy training protocols would be shortened, not only to reduce the amount of labor involved but in particular to allow the study of young mice, crucial for modeling confined neurodevelopmental periods. Furthermore, although mice may habituate to some extent to daily placement into operant chambers, even after repeated handling heart rate and corticosterone levels remained increased (Balcombe et al. 2004; Longordo et al. 2011) and the associated stress might impact on results (Hurst and West 2010).

Because of the limitations of currently available cognition assays, we here describe an automated home-cage task measuring initial response discrimination learning (DL) and reversal learning (RL) to assess cognitive flexibility within 4 d, without prior food deprivation and without human intervention. This task used an operant wall with three entry holes (CognitionWall) placed in an automated home cage (PhenoTyper) through which mice could navigate using both tactile and visual stimuli. Mice had to participate in the continuously running task to obtain food rewards in the absence of any other food which restricted the availability of food to periods of active task participation.

Reversal learning requires inhibition of an established response as well as the acquisition of a new response. Human subjects need substantially more trials to, and/or make more errors to, reach a given criterion during the reversal stage (RL) when compared with the initial discrimination stage (DL) (Dias et al. 1997; Tsuchida et al. 2010). To determine the translational validity of our task, DL and RL performance was compared in adult C57BL/6J mice, a common reference strain which is used for generating many mutant mouse models. Additionally, adolescent C57BL/6J mice were tested at 5 wk of age to establish the suitability of this task to assess cognitive flexibility during this early developmental stage.

We next tested whether the task provided sufficient complexity to detect deficits in associative learning in two mutant mouse models with well-established deficits in learning, i.e., transgenic mice overexpressing mutated human amyloid precursor protein and presenilin-1 (APPswe/PS1dE9; Jankowsky et al. 2004), a common mouse model for Alzheimer's disease (Reiserer et al. 2007; Cramer et al. 2012), and α-Calmodulin kinase II (αCaMKII) T305D mutant mice mimicking persistent inhibitory autophosphorylation of αCaMKII impairing learning and memory (Elgersma et al. 2002).

Finally, to establish whether this task measures similar processes underlying flexible stimulus-reinforcement learning in humans (construct validity), we interfered with orbitofrontal cortex neurocircuitry known to underlie this phenomenon (Cools et al. 2002; Hornak et al. 2004; Tsuchida et al. 2010).

## Results

**Figure 1.**
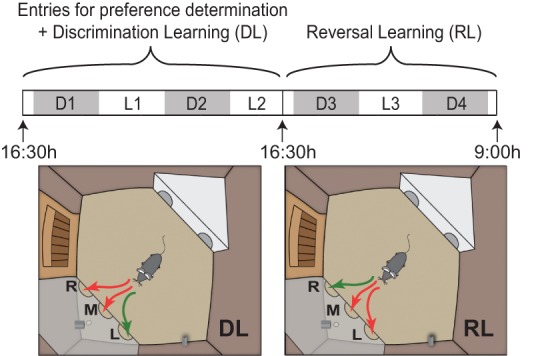
Schematic overview of the DL/RL task protocol in the home-cage PhenoTyper. The task started at 16:30 h, 2.5 h before the onset of the dark phase (19:00 h–7:00 h) when the CognitionWall was placed in front of the pellet reward dispenser. The dark phases are represented by the gray blocks (D1–4), the light phases by the white blocks (L1–3). The first entries through the CognitionWall were used to determine the initial preference for one of the three holes. Immediately thereafter, the discrimination learning stage started during which a left hole entry was rewarded at an FR5 schedule. Forty-eight h after the start of the task, the reversal learning stage commenced, during which a right hole entry was rewarded at an FR5 schedule. The CognitionWall with three holes, *right* (R), *middle* (M), and *left* (L), is shown, as well as correct (green) and incorrect (red) entrances during either DL or RL. The reward dispenser dropped food rewards behind the wall.

In the 4-d continuously running automated home-cage CognitionWall DL/RL task, mice first had to learn to earn food by going through the left hole in the CognitionWall that was placed in front of a pellet reward dispenser, i.e., the discrimination learning (DL) stage. After 2 d the right hole became rewarded, marking the start of the reversal learning (RL) stage (Fig. 1). These rewards were the only source of food for 4 d; however, mice were not limited in the number of rewards they could earn. In this task, mice were rewarded according to an FR5 schedule.

### C57BL/6J control mice reached performance criteria in DL and RL within 4 d

**Figure 2.**
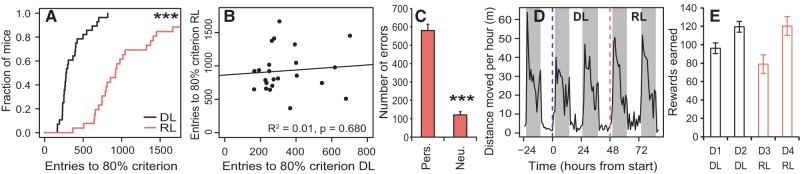
Task performance in adult C57BL/6J mice. (*A*) Kaplan–Meier plot of DL (*n* = 28) and RL (*n* = 26) performance of C57BL/6J mice. This plot shows the fraction of mice of that reached criterion (*y*-axis) at a given total number of entries (*x*-axis) during either DL or RL. The RL line does not reach a fraction of 1 because three mice did not reach the 80% performance criterion during RL. (*B*) Regression analysis of entries to 80% criterion in the DL stage and the RL stage in adult C57BL/6J mice. (*C*) Number of perseverative errors (*left* entry) and neutral errors (*middle* entry) during RL. (*D*) Average distance moved per hour during the 3rd day of the habituation protocol and the 4 d of the reversal learning protocol. The blue dashed line represents the start of the DL stage, the red dashed line represents the start of RL stage. (*E*) Number of rewards earned in the task per day of the task. (***) *P* < 0.001.

During DL, 25 of the 28 adult C57BL/6J mice reached the 80% performance criterion on day 1 of the task, on average 358 entries (±33) and 8.2 h ± 47 min after the start of the protocol, whereas the remaining three mice reached the criterion on day 2. Performance during the DL stage was not dependent on the initially preferred hole (*P* = 0.604^A^; for details on statistical tests, see Table 1). In line with data from human reversal learning tasks, mice required more entries (927 ± 68) to reach the 80% performance criterion during RL compared with DL (*P* < 0.001^B^) (Fig. 2A) when entering the other outside (right) hole was rewarded. Interestingly, there was no relationship between the number of entries to criterion during DL versus RL (Fig. 2B). Hence, DL performance did not predict RL performance. During RL, mice made more perseverative errors by going through the previously rewarded left hole, compared with neutral errors through the middle hole, before 80% criterion achievement (*P* < 0.001^C^)(Fig. 2C).

**Table 1.**
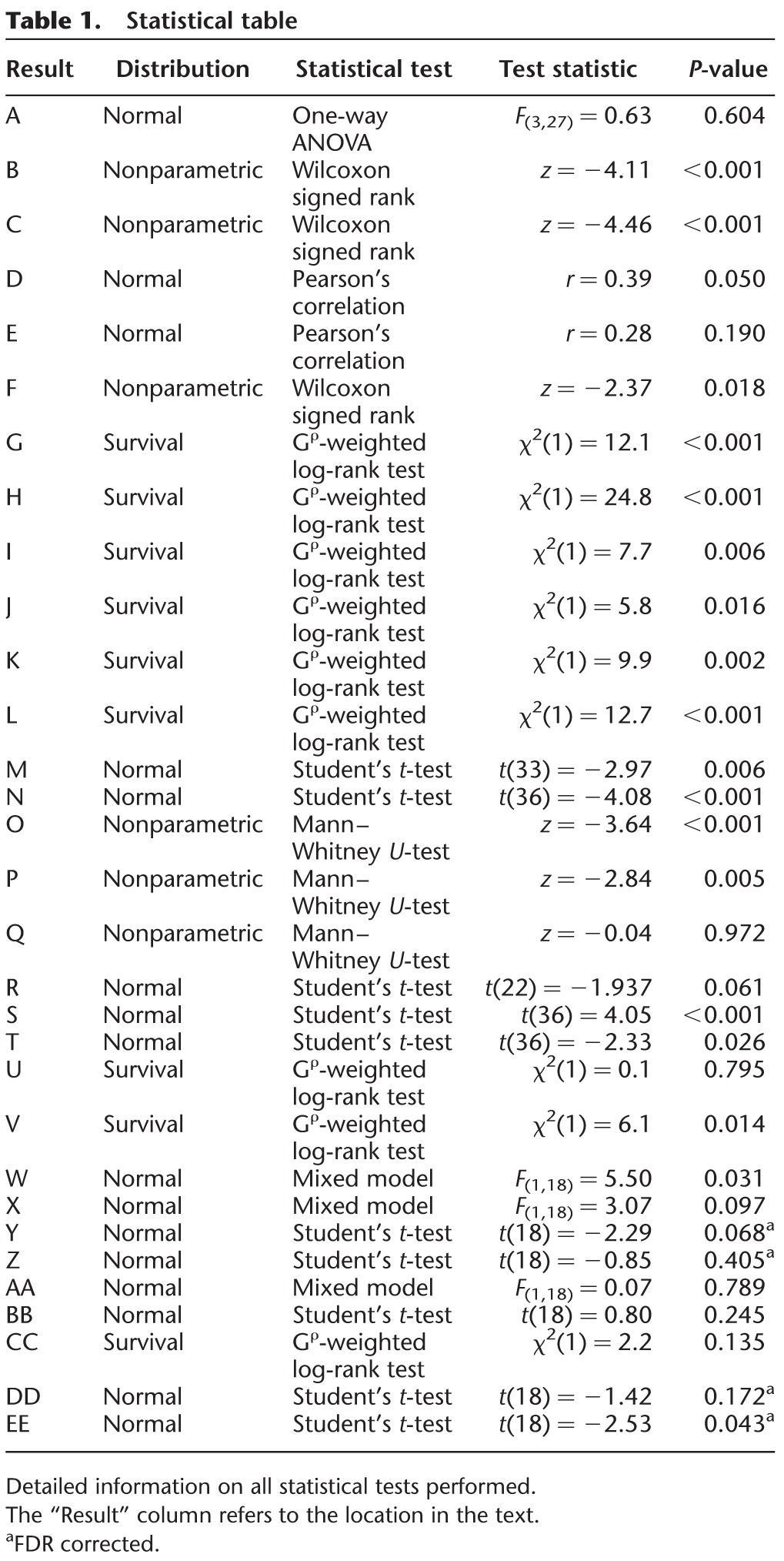
Statistical table

The average distance moved during the days of the task showed a similar circadian pattern to the day before the task (Fig. 2D) as well as to patterns previously observed in the PhenoTyper (Loos et al. 2014) and activity was predominantly limited to the dark phase, showing that this operant test in a home cage did not largely affect day/night rhythms.

Mice earned more pellets on the second day of each learning stage than on the first day (Fig. 2E) and earned on average 5.91 ± 0.33 g of rewards over the 4 d of the task. According to our standard procedure (see Materials and Methods), mice were fed additional pellets in case they did not earn enough to avoid potential weight loss. Only three of the 28 adult C57BL/6J mice received an additional 13 pellets on average after night two which led to a slight weight loss in the end of 3.2 ± 0.8% on average in all the mice. There was a weak, nonsignificant correlation between weight loss and the number of entries to criterion during DL (*r* = 0.38^D^) and RL (*r* = 0.28^E^).

Adolescent C57BL/6J mice that were tested at 5 wk of age were also successful in completing the DL/RL task within 4 d. Similar to adult C57BL/6J mice, adolescent mice required more entries to reach the RL criterion compared with the DL criterion (*P* = 0.018^F^). They earned on average 5.95 ± 0.55 g of rewards over the 4 d of the task, which led to an average weight loss of 3.2 ± 1%. Adolescent mice were not fed extra pellets.

Taken together, mice were able to complete the DL/RL task in 4 d. The absence of prior food deprivation combined with performance-dependent food availability and minimal extra feeding produced only a slight weight reduction compared with conventional operant protocols.

### Impaired discrimination learning in APP/PS1 and αCaMKII T305D mice

**Figure 3.**
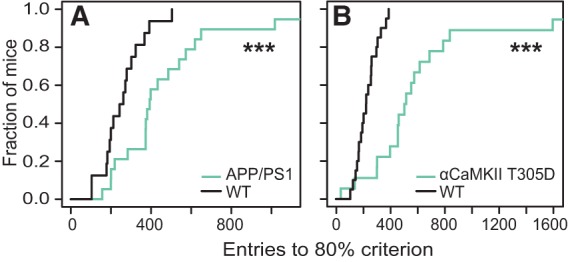
APP/PS1 and αCaMKII T305D mice show deficits in discrimination learning. (*A*) Kaplan–Meier plot of DL performance in APP/PS1 transgenic (*n* = 19) and their WT controls (*n* = 16). (*B*) Kaplan–Meier plot of DL performance in αCaMKII T305D mice (*n* = 18) and their WT controls (*n* = 20). (***) *P* < 0.001.

We next tested whether the task provided sufficient complexity to detect deficits in associative learning in two mutant mouse models with well-established deficits in learning. As predicted, APP/PS1 mice required substantially more entries to reach the 80% performance level during DL compared with their WT littermate controls (Fig. 3A) (*P* < 0.001^G^). Similarly, αCaMKII T305D mice also needed many more entries to criterion compared with their WT controls (Fig. 3B) (*P* < 0.001^H^). The learning impairment was consistently detected in two independent APP/PS1 batches (Batch 1: *P* = 0.006^I^; Batch 2: *P* = 0.016^J^), and in two αCaMKII T305D batches (Batch 1: *P* = 0.002^K^; Batch 2: *P* < 0.001^L^). These data demonstrate the sensitivity of the task to detect genotypic effects and the reproducibility among batches tested with several months interval.

The total number of entries during the task was higher in both mutants (APP/PS1: *P* = 0.006^M^; αCaMKII T305D: *P* < 0.001^N^) as well as the total distance moved during the task (APP/PS1: *P* < 0.001^O^; αCaMKII T305D: *P* = 0.005^P^). However, because performance was calculated as a moving average, this difference in activity was not underlying the observed impairment in discrimination learning in both mutant strains.

There was no difference in body weight between APP/PS1 transgenic mice and their WT controls before the test (*P* = 0.972^Q^). αCaMKII T305D mice were lighter than their WT controls (*P* < 0.001^R^). Mice with a lower body weight might require fewer food rewards to keep their body weight constant and might be less motivated to work for the food rewards in the task. However, αCaMKII T305D mice earned more rewards during the full 2 d of DL (*P* = 0.026^S^; Supplemental Fig. 1B) and APP/PS1 transgenic mice showed a trend toward more rewards earned (*P* = 0.061^T^; Supplemental Fig. 1A).

### OFC lesion: normal discrimination learning and disrupted reversal learning

To determine construct validity of the RL stage, we tested orbitofrontal cortex (OFC) ibotenic acid lesioned mice as this brain region is known to play an important role in animal and human reversal learning, but does not underlie discrimination learning.

**Figure 4.**
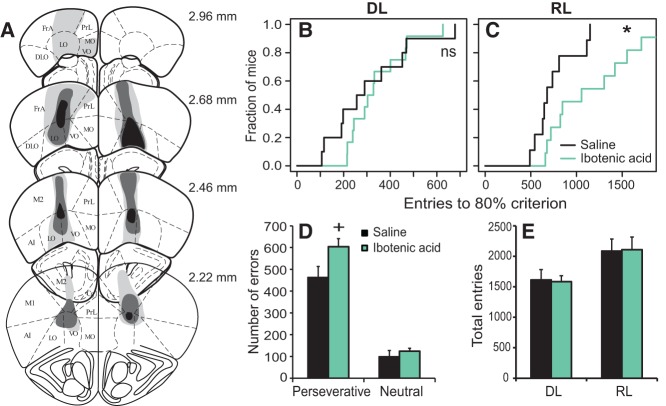
OFC lesions selectively impair reversal learning. (*A*) Distribution of lesioned areas, with the maximum extent of lesions denoted by light gray shading and the minimum extent of lesions by black shading. Representative affected areas, present in at least 50% of the mice, are shown in medium gray shading. No damage was observed in sections from the sham-lesion mice. Drawings were adapted from Paxinos and Franklin (2001), with permission of the publisher. (AI) agranular insular cortex, (Cg1) cingulate cortex, area 1, (DLO) dorsolateral orbital cortex, (FrA) frontal association cortex, (LO) lateral orbital cortex, (M1) primary motor cortex, (M2) secondary motor cortex, (MO) medial orbital cortex, (PrL) prelimbic cortex, (VO) ventral orbital cortex. (*B*) Kaplan–Meier plot of discrimination performance in ibotenic acid lesioned mice (*n* = 11) and their saline controls (*n* = 10). (*C*) Kaplan–Meier plot of reversal learning performance. (*D*) Number of perseverative and neutral errors made during the RL stage before reaching the 80% learning criterion for both ibotenic acid lesioned and saline control mice. (*E*) Total number of entries made during discrimination (DL) and reversal learning (RL) as a measure of general activity. (*) *P* < 0.05; ^+^*P* < 0.10.

Lesioned regions encompassed large areas of the lateral orbital and ventral orbital cortex, while leaving the medial orbital cortex and dorsolateral orbital cortex largely intact (Fig. 4A). Lesioned mice showed comparable numbers of entries to reach the 80% criterion compared with control saline-infused mice during the DL stage (*P* = 0.795^U^) (Fig. 4B). During the reversal stage, however, OFC lesioned mice required significantly more entries to reach the 80% performance criterion, and one mouse did not reach this criterion during the 2 d of the RL stage (Fig. 4C) (*P* = 0.014^V^).

This RL performance deficit in lesioned mice was mostly due to an increase in perseverative errors, whereas the number of neutral errors was comparable in the two groups (Fig. 4D) (main effect lesion: *P* = 0.031^W^; interaction lesion × error type: *P* = 0.097^X^; FDR corrected lesion effect on perseverative errors: *P* = 0.068^Y^; FDR corrected lesion effect on neutral errors: *P* = 0.405^Z^). Lesioned mice were similar to controls in general activity, as assessed by the total number of entries during both stages of the task (Fig. 4E) (Group effect: *P* = 0.789^AA^) and total distance moved (*P* = 0.245^BB^).

Exploring the performance of these mice at the start of the reversal learning stage showed a trend (*P* = 0.135^CC^) toward more entries to chance level performance (33% correct, calculated using a moving window) in OFC lesioned mice (Supplemental Fig. 2A). Before reaching chance performance levels, OFC lesioned mice made significantly more neutral errors (*P* = 0.043^DD^) and a trend toward more perseverative errors (*P* = 0.172^EE^; Supplemental Fig. 2B).

Thus, OFC lesions impaired reversal learning, but his became clearest when using a stringent performance criterion, while leaving discrimination learning and general activity unaffected.

## Discussion

This report presents an automated home-cage task for discrimination learning and reversal learning. To our knowledge, this is the first protocol that assesses discrimination learning as well as reversal learning in mice within (4) days without human intervention and the need for food deprivation, although leaving circadian activity patterns unaffected. The DL stage of the task showed to be complex enough to detect deficits in associative learning in mutant mice. In addition, the RL stage of the task showed both translational and construct validity with respect to human cognitive flexibility.

C57BL/6J mice required 1 d, and occasionally 2 d, to successfully acquire the discrimination between the three entry holes of the CognitionWall during the DL stage of the task. Reinforcing mice according to an FR5 schedule from the start of the DL stage onward, instead of starting with an FR1 and progressing to higher FR as typically done in operant tasks to facilitate acquisition of responding for reward (e.g., De Vries et al. 2005; Anderson et al. 2013), clearly did not hamper the acquisition of the initial discrimination, rather, it added complexity to this relatively simple learning task. In humans, probabilistic feedback is used to slow down the rate of learning in relatively simple learning tasks (Cools et al. 2002). In these tasks, correct responses are occasionally followed by negative feedback. Although the FR5 schedule we used does not directly compare with probabilistic reinforcement, the increase in difficulty of an FR5 schedule compared with an FR1 schedule probably contributed to the task's discriminative power.

The sensitivity of the task was assessed by testing two mouse models with well-established deficits in associative learning. Both batches of APP/PS1 transgenic mice and αCaMKII T305D mice showed considerable learning impairments in our protocol in terms of the number of entries required to reach the criterion. This consistent detection of a learning impairment in multiple batches of the same APP/PS1 transgenic and αCaMKII T305D mutant lines, tested with a period of several months in between, illustrates the robustness of the task. Nonetheless, we noticed differences in the number of entries required to reach criterion during discrimination learning among the wild-type and/or C57BL/6J groups of different experiments in this study, most likely due to differences in breeding conditions as well as genetic background. There is substantial evidence indicating that amyloid β oligomers adversely affect synaptic function in Alzheimer's disease (Haass and Selkoe 2007). Correspondingly, mimicking persistent inhibitory autophosphorylation of αCaMKII in the αCaMKII T305D mutant, previously shown to block long-term potentiation (Elgersma et al. 2002), led to a learning impairment in DL. Together, the DL impairment observed in both batches of both mouse models suggests that the DL stage of the task is suitable for detecting synaptic dysfunction in a sensitive and reproducible fashion.

In some learning paradigms, it is difficult to dissociate a difference in learning from general activity differences. Therefore, we analyzed the total number of entries needed to reach a criterion of 80% correct, computed as a moving window over the last 30 entries to assess learning in the DL/RL task. Since this performance measurement uses the fraction of correct over incorrect entries in the last 30 entries rather than the total number of entries or latency to reach criterion, this measurement is not likely to be influenced by general differences in activity between genotypes or groups. Hence, mice cannot achieve the learning criterion by only showing increased motor activity and making more entries.

The acquisition of the initial response discrimination (DL) and the subsequent reversal (RL) require the same set of motor responses, motivational states, and associative learning abilities. However, in line with previous reports in mice (Laughlin et al. 2011), the absence of a significant relation between entries to criterion during DL and RL suggests that different processes underlie performance during each stage of the task.

Human subjects need substantially more trials to, and/or make more errors to, reach a given criterion during the reversal stage (RL), when compared with the initial discrimination stage (DL) (Dias et al. 1997; Tsuchida et al. 2010). In addition, human subjects showed more perseverative errors compared with chance errors during reversal (den Ouden et al. 2013). We observed similar effects in C57BL/6J mice during the RL stage compared with DL stage: mice required more trials to criterion during RL compared with DL and they made more perseverative errors compared with neutral errors during RL, both establishing the translational validity of the task. In addition, these results suggest that this DL/RL task measures a form of cognitive flexibility involving response inhibition (Izquierdo and Jentsch 2012; Bari and Robbins 2013).

The role of the orbitofrontal cortex in the RL stage of the DL/RL task substantiates the construct validity of the task. The OFC is known to underlie flexible stimulus-reinforcement learning in humans (Cools et al. 2002; Hornak et al. 2004; Tsuchida et al. 2010) and, similar to other animal studies (Hamilton and Brigman 2015), we observed that the OFC plays a fundamental role in reversal learning. However, the effect of our lesions on perseverative errors was only limited which asks for further investigation of the role of the OFC in response inhibition.

Lesion sites seemed to encompass small areas of the frontal association cortex (FrA). To our knowledge, the FrA has not been implicated in reversal learning. Nonetheless, a recent study showed that the FrA is important for stimulus integration in association learning (Nakayama et al. 2015). Since initial discrimination learning was not affected in our lesioned animals, we did not expect a significant contribution of lesions of the FrA to the reversal learning deficit in the lesioned animals.

Conventional operant procedures require daily periods of total food deprivation until the next feeding moment to maintain 80%–90% of their original body weight (Garner et al. 2006; Mar et al. 2013). In the described task, the availability of food is task performance dependent. However, there is no restriction on the amount of rewards mice can obtain. To our knowledge, we are the first to report such a food availability schedule. This procedure required only limited additional feeding, mostly due to some reward dispenser errors, and prevented substantial weight loss. Whether the motivation to perform an operant task differs between the present food regime (working for all food) and a classical regime (being on a 90%–80% diet and working for extra) will require systematic follow-up studies. Most important, the present regime presumably reduced stress induced by conventional food deprivation schedules, which was previously reported to interact with cognitive performance (Cabib et al. 2000; Papaleo et al. 2012).

Remmelink et al. (2015) showed that appetitive operant testing for food rewards is possible in the presence of standard chow. However, mice also engaged in many other behaviors besides the food-motivated learning responses during that task. This limited the power to detect learning differences between groups of mice. The current results show that removing access to standard chow generates sufficient motivation for mice to learn the contingencies of food acquisition, preserving the sensitivity to detect differences in learning abilities between mice, while only slightly affecting bodyweight. Furthermore, the task did not largely affect day/night rhythms. This suggests that the task capitalizes on natural foraging behavior during specific times of the day.

Two aspects most likely have contributed to the short duration of the task. First of all, because of continuous testing, the achieved training intensity per training day is substantially higher compared with conventional operant tasks as well as maze-based tasks. The lack of a retention period in between training sessions, as present in other tasks, may also have affected learning rate as described previously (Smolen et al. 2016) and thereby the duration of the task. However, given the difference in nature of the present task and “conventional” tasks, it is difficult to draw conclusions on the effect of the lack of a retention period in between training sessions on learning rates. Second, since the task used location-based information with tactile as well as visual cues rather than pure visual stimuli (such as used in touchscreen based tasks), the task's demands came closer to what mice evolutionarily are well adapted for, which likely increased the speed of learning.

Overall, the short duration of the task allows for high-throughput assessment of mouse cognition. Previous studies also showed that measures of learning can be collected in standardized automated home-cage environments without human interference (Galsworthy et al. 2005; Mechan et al. 2009; Gallistel et al. 2010; Endo et al. 2011; Balci et al. 2013; Puścian et al. 2014; Remmelink et al. 2015); however, all these tasks restricted reinforcement schedules to specific times of the day. We here demonstrate that continuous testing is compatible with operant testing which allowed for the short duration of the reversal learning protocol as well as animal paced task participation.

The automation achieved with the home-cage DL/RL task also significantly reduced human labor. More importantly, human interference is known to influence task outcome (Crabbe et al. 1999; Sorge et al. 2014) and can induce stress in mice (Balcombe et al. 2004; Hurst and West 2010). The fact that this new task avoids this, may improve the replicability of cognitive testing as well as animal welfare.

Although the current reversal learning protocol in home cages only takes 4 d whereas conventional tasks in operant boxes may take up to 40 d to complete (Mar et al. 2013), conventional tasks come with the advantage that multiple subjects can be trained in the same box on a given day. Hence, whether home cages have a higher or lower screening capacity compared with conventional operant boxes depends on the exact duration of the protocols, the number of available boxes and the number of subjects that need to be tested per day. Regardless of screening capacity, home-cage testing reduces labor and increases standardization of the test environment.

Whereas in conventional tests it is possible to socially house mice in between training sessions, during the multiple-day home-cage testing in our setup mice were continuously housed individually. Individual housing of mice is generally considered undesirable, although there is an active debate in literature on the effect of individual housing of male mice. Indeed, even though group-housed male mice perform aggressive behavior to develop and maintain social hierarchy, both dominant and subordinate male mice appear to prefer the proximity of another male over individual housing (Van Loo et al. 2004). Yet in contrast, Kamakura et al. (2016) reported lower corticosterone levels in individually housed male mice compared with group-housed animals. Moreover, Arndt et al. (2009) reported that neither male nor female mice that were housed individually showed stronger signs of stress than their socially housed counterparts. Whether or not individual housing contributed to distress, individual housing during our test precluded an effect of hierarchy on task performance and/or competition for food and water reward as may be the case for home-cage settings using social housing (e.g., IntelliCages; Galsworthy et al. 2005; Mechan et al. 2009; Endo et al. 2011). Additionally, individual housing allows for a voluntary drug administration procedure as described previously (Aarts et al. 2015).

In conclusion, cognitive performance of mice can be studied in an automated and continuous fashion with comparable task sensitivity as previous tests. We circumvented labor-intensive and stress-inducing food restriction regimes and substantial weight loss by making food availability performance dependent. These improvements substantially enhance the efficiency of measuring associative learning and cognitive flexibility in mice, and improve animal welfare. In addition, they provide a valuable solution for drug testing and, due to its short duration, the study of cognition during confined neurodevelopmental periods.

## Materials and Methods

### Mice

C57BL/6J breeding pairs were obtained from Charles River Laboratories (L'Arbresle, France; European supplier of Jackson Laboratories) and bred in-house in individually ventilated cages (IVCs) for a maximum of three generations. Thirty-one male adult mice were tested at 11 wk of age. In addition, nine male adolescent mice were tested at 5 wk of age. Data of five mice were fully removed from the analysis (three adults, two adolescents), and two adult mice were removed from analysis of the reversal stage, due to pc or reward dispenser errors.

Eighteen WT and 22 transgenic male mice of an APPswe/PS1dE9 (APP/PS1) (Jankowsky et al. 2004) colony that had initially been backcrossed for more than 10 generations to a C57BL/6OlaHsd background and was backcrossed to C57BL/6J for two to three generations were bred in IVCs and tested in two batches (Batch 1: 11 WT, 14 Tg; Batch 2: 7 WT, 8 Tg) at 6–7 mo of age, with 2 mo in between the two batches. Data of two WT and two Tg APP/PS1 mice of Batch 1 and one Tg APP/PS1 mouse of Batch 2 were removed from the analysis because of pc or reward dispenser errors.

The αCaMKII T305D mutant line (Elgersma et al. 2002) had been maintained for more than 10 generations on a C57BL/6J background. Twenty-two WT and 18 homozygous mutant male mice (Mut) were bred in conventional open cages and were tested in two batches at 19 wk of age (Batch 1: 16 WT, 9 Mut; Batch 2: 6 WT, 9 Mut), with a 6 mo period in between batches. All mutant mice tested were compared with their wild-type littermates. Data of two WT αCaMKII T305D mice from Batch 1 were removed from the analysis because of pc or reward dispenser errors.

For the orbitofrontal cortex lesion experiment, 30 C57BL/6J mice were obtained from Charles River Laboratories at 9 wk of age. After shipment, they were allowed to acclimate to their novel environment for 2 wk before surgery was performed. Data of seven mice were removed from the analysis because of pc or reward dispenser errors.

At least 1 wk before the experiment, mice were single housed on sawdust in standard Makrolon type II cages enriched with cardboard nesting material, with water and food (2018 Teklad, Harlan Laboratories) ad libitum (7:00/19:00 lights on/off; providing an abrupt phase transition). Mice were removed from analysis when hardware errors occurred. All experiments were carried out in accordance with the European Communities Council Directive of 24 November 1986 (86/609/EEC), and with approval of the Animal Experiments Committee of the VU University.

### Orbitofrontal cortex lesions

In 15 mice, bilateral lesions were made in the orbitofrontal cortex (OFC) (anteroposterior +2.55 mm, lateral +1.10 mm, ventral +2.60 mm with respect to Bregma) by injecting 0.3 μL ibotenic acid dissolved in phosphate-buffered saline (PBS) with an injection rate of 0.1 μL/min during stereotactic surgery under deep anesthesia (1.2% freshly prepared avertin, 0.02 mL/g body weight, intraperitoneal (i.p.) injection) and after s.c. injecting 0.01 mg/kg of the analgesic compound buprenorphine (Temgesic, RB Pharmaceuticals Limited). Sham-lesioned mice (*n* = 15) underwent the same procedures as the ibotenic acid-infused mice, except that 0.9% sterile NaCl was infused. Mice were allowed to recover for 7–11 d before the start of the behavioral experiment. After the experiment, mice were intracardially perfused with 4% paraformaldehyde in PBS. Their brains were removed, stored at 4°C in PBS with 30% sucrose for three nights. Hereafter, they were sectioned coronally on a cryostat at −14°C with a section thickness of 25 μm and collected in PBS. Sections were stained with Cresyl violet to visualize Nissl bodies, mounted on glass slides, and inspected for lesion size and location using a light microscope. One mouse that did not show a lesion and was removed from the analysis.

### Automated home cage

The PhenoTyper (model 3000, Noldus Information Technology) is an automated home cage in which behavior was tracked by video and in which hardware actions were triggered by the location of the mouse (Maroteaux et al. 2012). The cage was equipped with a drinking bottle, feeding station, and a triangular-shaped shelter with two entrances in one corner. In the opposite corner, an aluminum tube of a reward dispenser protruded into the cage. For the reversal learning task, an opaque Perspex wall with three holes was placed in front of the reward dispenser (Fig. 1) (CognitionWall; H = 25 cm, W = 17 cm, Diameter holes = 3.3 cm). White α cellulose bedding material was supplied in the cage (ALPHA-Dri, Shepherd Specialty Papers Inc. ), providing high contrast with dark-colored mice under infrared light. Approximately 2–3 cm bedding material was used, sufficient for housing of individual mice for up to 2 wk without bedding change. For the experiments described here, 44 PhenoTypers were available located in a dedicated testing room, which allowed for parallel testing of experimental and control groups. Cages were washed with water and detergent in between experiments.

### Discrimination- and reversal learning (DL/RL) task protocol

Mice were introduced to a PhenoTyper during the light phase (14:00–16:00 h) and housed in this cage without any further human handing for the next 7 d. Water was provided ad libitum during the entire period, and ad libitum food was available in the feeding station for the first 3 d.

Fifteen minutes before the start of the discrimination learning and reversal learning (DL/RL) task at 16.30 h on the 3rd d in the Phenotyper, the CognitionWall was placed in front of the reward dispenser spout (see Fig. 1). After placement, one free reward was dispensed and standard chow was removed from the feeding station. The entries made through the wall, until one of the three holes had an entry count of 10 or higher and the entry count of the left hole was a multiple of 5, were used to determine whether mice displayed an initial preference for one of the three holes. During these entries no rewards were dispensed.

Thereafter, mice had to learn to earn their food (Dustless Precision Pellets, 14 mg, Bio-Serve) by going through the left hole in the wall (Discrimination Learning, DL) for the next 2 d (DL1 and DL2). The middle and right holes were deemed incorrect holes and passing through these holes did not have any consequences. During the subsequent 2 d, the rewarded hole was switched to the right hole (Reversal Learning: RL1 and RL2). During DL and RL, one reward was delivered for every fifth entry through the correct hole (FR5 schedule of reinforcement). Mice were not required to make five consecutive correct entries, i.e., no chaining requirement. The FR5 schedule was chosen after an initial pilot experiment showed that an FR1 ratio resulted in satiety, as indicated by accumulation of nonconsumed rewards in the cage.

Online display of the number of earned rewards allowed the experimenter to evaluate food intake during the experiment. In a pilot experiment, we quantified that C57BL/6J mice required around 100 food rewards to maintain body weight. Therefore, during the task when mice earned fewer than 100 rewards per day for two or more consecutive days, mice we fed extra reward pellets at the beginning of the light phase to reach 100 pellets.

### Data analysis

The total number of entries needed to reach a criterion of 80% correct, computed as a moving window with window size 30 (i.e., 24 correct entries of the 30 last entries), was used as a measure of learning during DL and RL. To empirically test whether mice can reach this learning criterion by chance, we simulated a total number of 2000 entries and simulated chance levels of entries through the left, right, and middle hole of 40%, 20%, and 40%, respectively (numbers and percentages are based on actual performance of mice in the experiments described in this paper). In 10 analyses of 1000 permutations, on average 6.5 ± 0.58 mice (0.006%) reached the learning criterion of 80% using the moving window of the size of 30 entries. Thus, it is not conceivable that any mouse will reach the learning criterion by chance. Differences in performance between groups (genotype/lesion) were assessed using the G^ρ^-weighted log-rank test for differences between two or more Kaplan–Meier survival curves.

During the reversal stage, the number of entries through the previously correct left hole provided a measure of perseverative errors. The number of entries through the never-rewarded middle hole represented a measure of neutral errors (Arnsten et al. 1997; Jentsch et al. 2002; Lee et al. 2007). The total number of entries and the total distance moved were taken as measures of general activity. These measures were statistically analyzed using independent or paired *t*-tests, dependent on whether the parameter was measured repeatedly. If the data did not meet the assumption of normality, a nonparametric alternative was used, i.e., Mann–Whitney *U*-test or Wilcoxon signed-rank test. A factorial repeated measures ANOVA was used to compare the different types of errors between groups, followed by post hoc tests per error type that were FDR corrected for multiple testing. Variation is presented as the SEM. Test results are considered significant at *P* < 0.05.

## Competing interest statement

For full transparency, E.R. and M.L. are full time employees of Sylics (Synaptologics BV), a private, VU University spin-off company that offers mouse phenotyping services including the described task. A.B.S. and M.V. participate in a holding that owns Sylics shares and have received consulting fees from Sylics.

## Supplementary Material

Supplemental Material
