# Supplementary material for: Measuring discrimination- and reversal learning in mouse models within 4 days and without prior food deprivation
Source: Learn Mem. 2016 Nov;23(11):660–7. doi: 10.1101/lm.042085.116 (PMC5066605; doi:10.1101/lm.042085.116)
Supplement: Supplemental Material [file supp_23_11_660__index.html]

Supplemental Material 

# Measuring discrimination- and reversal learning in mouse models within 4 days and without prior food deprivation

## Supplemental Material

**Files in this Data Supplement:**

- Supplemental\_Materials.pdf
