# Supplementary material for: Measuring discrimination- and reversal learning in mouse models within 4 days and without prior food deprivation
Source: Learn Mem. 2016 Nov;23(11):660–7. doi: 10.1101/lm.042085.116 (PMC5066605; doi:10.1101/lm.042085.116)
Supplement: Supplemental Material [file supp_23.11.660_Supplemental_Materials.pdf]

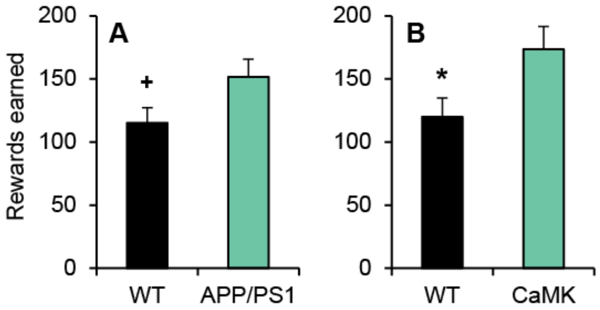

Supplementary figure 1 | Number of rewards earned during DL. (A) There was a trend towards more rewards earned during the two days of the DL stage in APP/PS1 mice ($^{+}$p = 0.061). **(B)** αCaMKII T305D mice earned more rewards than WT controls (*p = 0.026). Mean ± SEM.

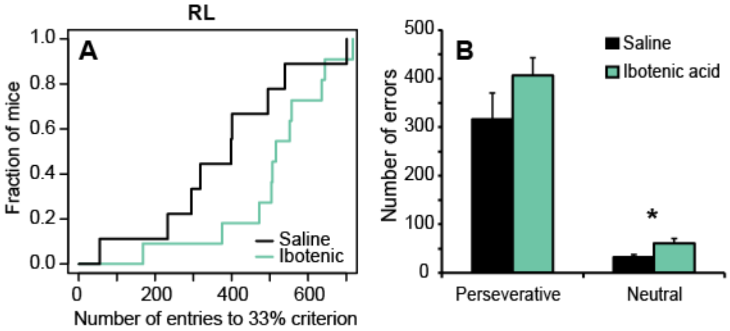

Supplementary figure 2 | Effect of OFC lesions on reversal performance to chance level (33%). (A) There was a trend towards more entries to 33% criterion in OFC lesioned mice (p = 0.135). **(B)** Errors made before mice reached the 33% performance criterion. Effect of lesion (p = 0.109); Lesion effect on perseverative errors (p = 0.172, FDR corrected); Lesion effect on neutral errors (p = 0.042, FDR corrected). Mean ± SEM.
